# Spark-Discharge-Activated 3D-Printed Electrochemical Sensors

**DOI:** 10.1021/acs.analchem.4c01249

**Published:** 2024-06-13

**Authors:** Juan F. Hernández-Rodríguez, Maria G. Trachioti, Jan Hrbac, Daniel Rojas, Alberto Escarpa, Mamas I. Prodromidis

**Affiliations:** † Department of Analytical Chemistry, Physical Chemistry and Chemical Engineering, University of Alcalá, Alcalá de Henares 28802, Madrid, Spain; ‡ Department of Chemistry, University of Ioannina, 45 110 Ioannina, Greece; § Department of Chemistry, Masaryk University, 625 00 Brno, Czech Republic; ∥ Chemical Research Institute “Andres M. Del Rio”, University of Alcalá, Alcalá de Henares 28802, Madrid, Spain

## Abstract

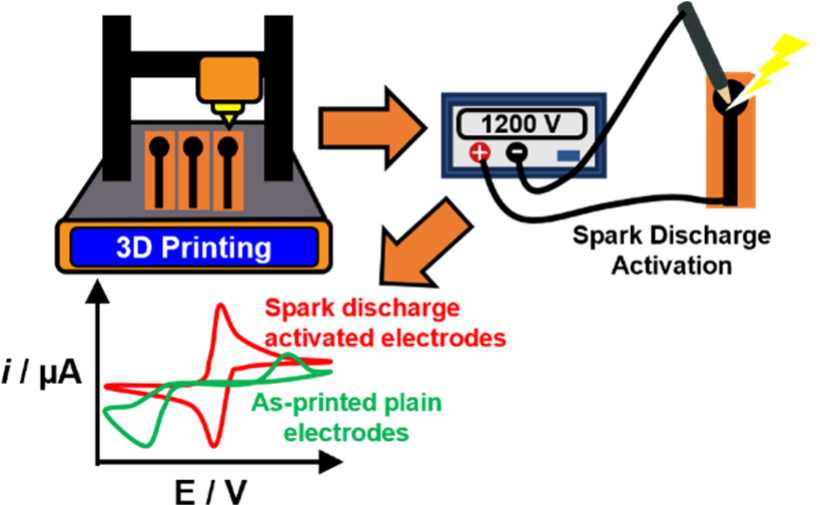

3D printing technology is a tremendously powerful technology to fabricate electrochemical sensing devices. However, current conductive filaments are not aimed at electrochemical applications and therefore require intense activation protocols to unleash a suitable electrochemical performance. Current activation methods based on (electro)chemical activation (using strong alkaline solutions and organic solvents and/or electrochemical treatments) or combined approaches are time-consuming and require hazardous chemicals and dedicated operator intervention. Here, pioneering spark-discharge-activated 3D-printed electrodes were developed and characterized, and it was demonstrated that their electrochemical performance was greatly improved by the effective removal of the thermoplastic support polylactic acid (PLA) as well as the formation of sponge-like and low-dimensional carbon nanostructures. This reagent-free approach consists of a direct, fast, and automatized spark discharge between the 3D-electrode and the respective graphite pencil electrode tip using a high-voltage power supply. Activated electrodes were challenged toward the simultaneous voltammetric determination of dopamine (DP) and serotonin (5-HT) in cell culture media. Spark discharge has been demonstrated as a promising approach for conductive filament activation as it is a fast, green (0.94 GREEnness Metric Approach), and automatized procedure that can be integrated into the 3D printing pipeline.

## Introduction

3D printing technology is a tremendously powerful technology to fabricate electrochemical sensing devices. Specifically, fused filament fabrication (FFF) is gaining much attention these years to fabricate customized electrochemical sensors.^1−3^ FFF multimaterial printing makes it possible to fabricate complex platforms with integrated electrodes in a single fabrication step,^4−6^ and it also allows electrode arrangements that are not possible to accomplish with conventional electrode fabrication technologies.^7,8^ Traditional technologies require specialized equipment and expertise, which are not accessible to every laboratory and hence inhibit new developments in this area.^9^ On the contrary, FFF is low-cost equipment that can be installed in most laboratories. More importantly, the whole fabrication process is automatized and integrated in a single process, and it requires no operator intervention. For these reasons, FFF is a promising technology for fast prototyping and laboratory-scale fabrication of miniaturized electroanalytical devices.

Commercial conductive filaments are composed of a thermoplastic support, mainly poly(lactic acid) (PLA) and a carbon filler that provides the electrical conductivity properties. Carbon black, graphene, and carbon nanotubes stand out among the carbon fillers. Despite being conductive, as-printed electrodes are not suitable for most electrochemical applications; hence, their surface needs to be activated. Conductive filament activation is a hot topic these days, and there are plenty of strategies proposed for this end at different stages of the fabrication: pre-,^10^ mid-,^11^ and postprinting. Postprinting methods are the most extended because they can be performed on-demand. The different activation strategies aim to eliminate the thermoplastic material from the surface of the electrode, uncovering the conductive carbon material. Chemical treatment either dissolves completely the polymer by immersing electrodes in organic solvents^12,13^ or saponificates the polymer chains in alkaline media.^10,14^ Other popular methods include electrochemical treatments,^11,15,16^ surface sanding,^17^ laser scribing,^18^ or oxygen plasma.^19^ Some of these approaches are not effective on their own and require to be combined to obtain a suitable response.^20^ Besides, many of these approaches are time-consuming and require operator intervention. Therefore, fast and automatized activation approaches performed during printing are urgently needed for the advance of this technology.

In this sense, spark discharge, which has been recently proposed as an ecofriendly, reagentless, and direct method for the *in situ* modification of screen-printing electrodes (SPE), arises as a suitable method for the electrochemical activation of conductive 3D printing surfaces. In this approach, sponge-like graphite and low- dimensional graphite flakes from pencil lead tip electrodes^21^ or gold nanoparticles from gold tip electrodes^22,23^ have been demonstrated to be deposited onto the screen-printed working electrodes upon the direct spark discharge between the SPEs and the respective tip electrodes using a common high-voltage power supply. Unlike the currently conductive filament activation methods explored, the spark-discharge process is extremely fast (ca. 30 s per electrode) and automatized by programming a high-voltage supply and an Arduino-operated 3D positioning device. This way, the speed, frequency, and position of the sparks can be precisely controlled, hence improving the reproducibility of the technique. Besides, it is a green procedure as it involves no solvents or other chemicals, and it is carried out at ambient conditions.^22^

In this Technical Note, the suitability of conductive filament activation by spark discharge from pencil leads was studied. First, the effect of the spark-discharge treatment was studied in electrodes of varying thickness, as it has been reported that electrochemical performance improves with the thickness.^24,25^ Following this, the electrode surfaces underwent characterization by using scanning electron microscopy (SEM), attenuated total reflectance infrared spectroscopy (ATR-IR), and Raman spectroscopy. Results revealed polymer degradation, a slight reduction in surface defects, an increase in the graphitic domain/amorphous carbon ratio, and the presence of sponge-like and low-dimensional nanostructures post spark discharge. Additionally, spark-discharge activation significantly reduced the impedance magnitude of the electrodes, as made evident by electrochemical impedance spectroscopy (EIS) measurements. Finally, the spark-discharge-activated 3D-printed carbon electrodes were challenged toward the simultaneous determination of dopamine (DP) and serotonin (5-HT) in spiked cell culture medium.

## Experimental Section

### Materials and Reagents

Graphite pencil (Castell 9000, 2B hardness) was a product of Faber–Castell. Potassium hexacyanoferrate(III) was purchased from AnalaR, while potassium hexacyanoferrate(II) trihydrate was from Merck. Dopamine hydrochloride, serotonin hydrochloride, Dulbecco’s modified Eagle’s medium (DMEM), and all the other reagents were from Sigma-Aldrich. Double distilled water was used throughout.

### Apparatus

Electrochemical characterization measurements were conducted with an AUTOLAB PGSTAT12/FRAII electrochemical analyzer (Metrohm Autolab BV, The Netherlands) by using a conventional three-electrode electrochemical cell. Plain or sparked 3D-printed carbon electrodes of 3 mm diameter were used as the working electrode, a Pt-wire as the auxiliary electrode, and a Ag/AgCl, 3 M KCl electrode (IJ Cambria) as the reference electrode. Cyclic voltammograms (CVs) were conducted in 0.1 M KCl, pH 3, containing 1 mM potassium hexacyanoferrate(III) at a scan rate of 0.020 V s^–1^. EIS studies were performed in a mixture of 1 + 1 mM potassium hexacyanoferrate(II)/(III) in 0.1 M PBS, pH 7, over the frequency range from 100 kHz to 0.1 Hz by using a sinusoidal excitation signal of 0.010 V (rms) superimposed on 0.3 V DC potential.

SEM images were taken with the Phenom Pharos G2 Desktop FEG-SEM (Thermo Fisher Scientific) on Cr sputtered specimens (Q150T ES Plus sputter coater, Quorum Technologies Ltd.). ATR-IR was conducted with a Spectrum Two instrument (PerkinElmer, United States). Spectra were collected as the average of 16 scans with a resolution of 4 cm^–1^ from 400 to 4000 cm^–1^. Raman spectra were obtained with a 532 nm wavelength with laser power set at 0.28 mW at 50× magnification using a fully integrated confocal Raman microscope (Labram SoleilTM, Horiba Scientific). The Raman spectra were collected in the 100–3000 cm^–1^ range. The integration time was 100 s, with three accumulations.

### Fabrication of the 3D-Printed Electrochemical Sensors

The 3D-printed electrochemical sensors were fabricated by employing a single extruder Prusa i3MK3S+ (Prusa Research, Czech Republic) with a 0.4 mm nozzle. Electrodes were printed with a PLA-CB filament (Protopasta CDP11705, Protoplant, Canada) and the insulating parts in poly(ethylene terephthalate glycol) (PETg) (Smart Materials, Spain). Filaments were changed manually at each layer, and a cold pull cleaning procedure was performed to eliminate any residues of PLA-CB from the nozzle.^5^

Devices were printed at 25 mm s^–1^ with 100% infill, and nozzle and bed temperatures were set to 230 and 90 °C, respectively. The layer height was set to 0.1 mm, and the total number of layers ranged from 1 to 10 layers. To prevent the filaments from absorbing moisture, they were kept in a filament dryer at 45 °C.

### Treatment of the Electrode Surface with Spark Discharge

The treatment of 3D-printed carbon electrodes (1, 3, 5, 6, 8, 10 layers using 0.1 mm layer height) by spark discharge was performed at 1.2 kV at ambient conditions in the presence of an external capacitor (5.3 nF) connected in parallel with the terminals of a high-voltage power supply. The graphite electrode tip (2B pencil), connected as the (−) pole, and the 3D-printed carbon electrode, connected as the (+) pole, were brought in proximity (<1 mm) until the spark discharge occurred. The sparking process was conducted through a “linear” mode (20× parallel lines at a speed 100 mm/min) with the aid of a G-code controlled 2D-positioning device. The distance between the sparking lines was set to be 0.15 mm to ensure an even distribution of the sparking lines across the 3 mm diameter (active) electrode surface. The treatment of each electrode by spark discharge was conducted twice, and the total duration of the sparking process was 30 s. Details on the experimental setup for the treatment of electrodes with spark discharge are given in refs (22) and (26).

### Simultaneous Determination of Dopamine and Serotonin

Square wave (SW) voltammetry was conducted for the analytical application employing an EmStat4s (Palmsens BV, The Netherlands) portable potentiostat. Sparked 3D-printed carbon electrodes were immersed in standard solutions containing either DP, 5-HT, or a mixture of both analytes and the supporting electrolyte (0.1 M PBS, pH 7). Electrodes were scanned from 0 to 0.65 V, employing a waveform of 0.05 V amplitude, 5 Hz frequency, and 5 mV step. Before measurements, each electrode was scanned in the supporting electrolyte five times to stabilize the signal and clean the surface.

The concentrations of DP and 5-HT were calculated by registering the peak currents at ca. +0.16 and +0.35 V, respectively. Spiked cell culture samples were prepared by diluting concentrated standard solutions of DP and 5-HT prepared in PBS in commercial cell culture media (DMEM).

## Results and Discussion

### Design and Electrochemical Characterization of Sparked 3D-Printed Carbon Electrodes

**Figure 1:**
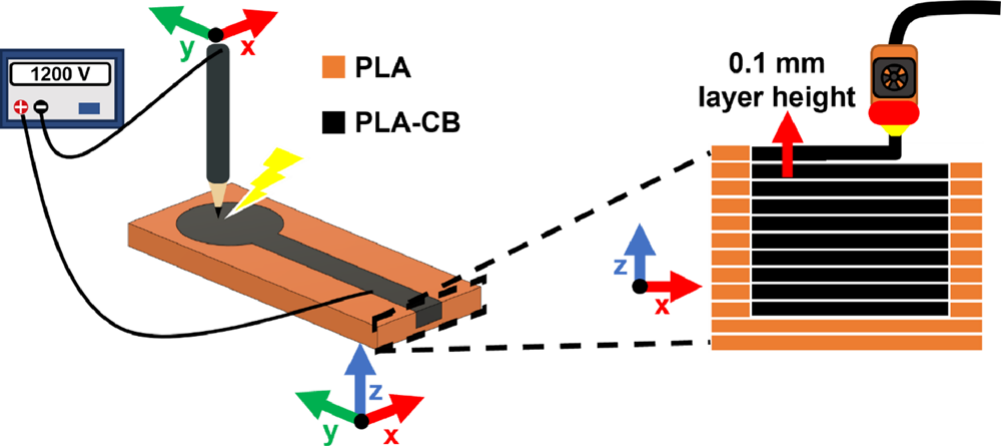
Schematics for the fabrication and activation of a sparked, 3D-printed carbon electrode. The zoomed area on the right shows a cross section of the electrode with the printed layers.

In the fabrication of 3D-printed carbon electrodes, multiple parameters can be adjusted, which have a significant contribution in their electrochemical performance.^24,27,28^ In contrast to electrodes fabricated with other techniques, the height of the FFF can be easily set by varying the number of printed layers. In this work, we propose a 3D-printed electrode design using different layers, as schematized in Figure 1.

**Figure 2:**
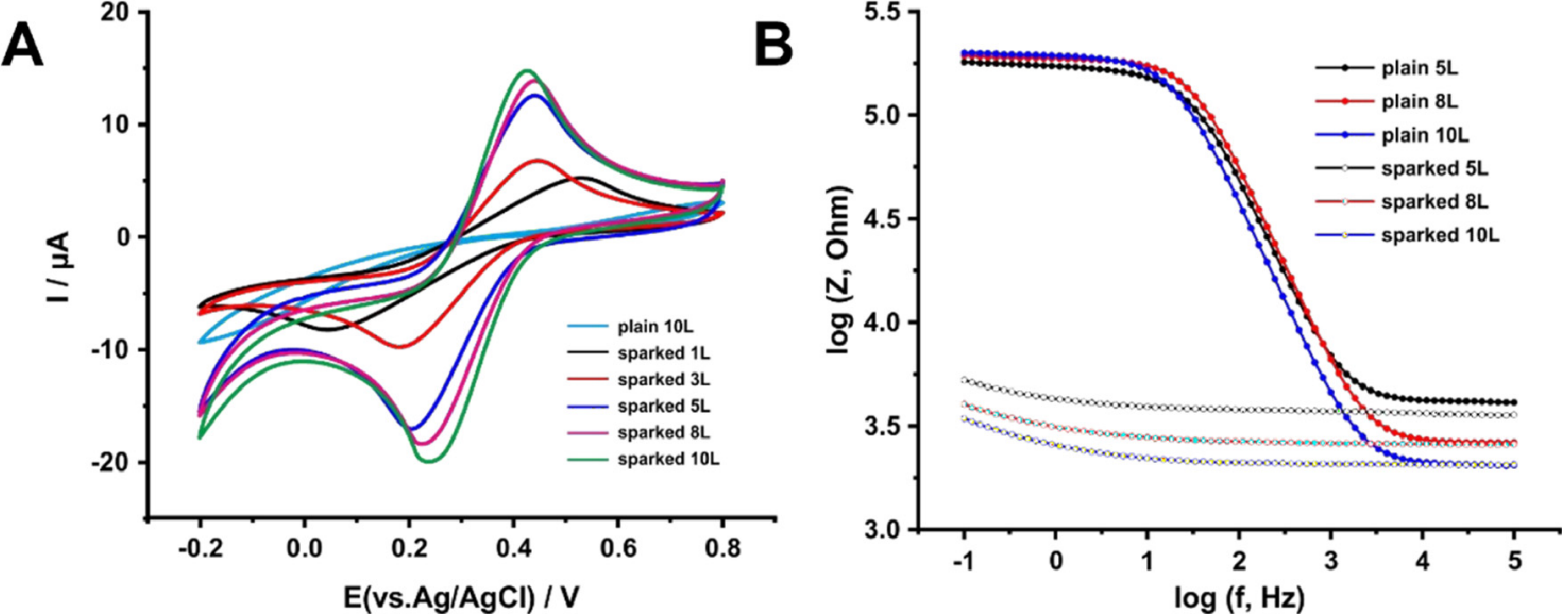
Electrochemical characterization of the sparked 3D-printed carbon electrodes. (A) CVs of plain (10L) and sparked (1L, 3L, 5L, 8L, and 10L) 3D-printed carbon electrodes in 0.1 M KCl, pH 3.0, containing 1 mM potassium hexacyanoferrate (III). Scan rate: 20 mV s^–1^. (B) Bode magnitude plots of plain (5L, 8L, and 10L) and sparked (5L, 8L, and 10L) 3D-printed carbon electrodes in 0.1 M PBS, pH 7, containing 1 + 1 mM potassium hexacyanoferrate (II)/(III).

Then, the electrochemical performance of plain and sparked 3D-printed carbon electrodes with different thicknesses (ranging from 0.1 to 1 mm, 1–10 layers (L)) was examined by performing CV measurements toward potassium hexacyanoferrate (III), which was used as a model inner-sphere redox probe. CVs are illustrated in Figure 2A and show that plain 3D-printed carbon electrodes exhibited poor electrocatalytic properties. Like the response observed for the plain 10-layer (10 L) electrode, all of the other plain electrodes also gave featureless CVs (data not shown). In contrast, sparked 3D-printed carbon electrodes exhibited a thickness-dependent improvement of their electrocatalytic properties, resulting in CVs that possessed well-defined redox peaks with different voltage–current characteristics. The effect of the different printing layers on the electroactive surface area (*A*), the heterogeneous electron transfer rate constants (*k*^0^), the peak potential separation (Δ*E*_p_) values, and the current densities of the respective electrodes are shown in Table S1. Data demonstrate that the penetration of the spark process is quite sufficient and enabled the effective removal of the dielectric PLA from the filaments even at the thicker electrodes, thus resulting in a gradually increased amount of graphite exposure at the electrode/electrolyte interface as the thickness of the electrodes increased. Indeed, thicker electrodes exhibited higher electroactive surface area, current densities, heterogeneous electron transfer rate constants, and lower Δ*E*_p_ values, demonstrating enhanced electrocatalytic properties. The 10L electrode gave the best electrocatalytic properties in terms of Δ*E*_p_, peak current density, area, and *k*^0^, as shown in Figure 2A and Table S1.

Furthermore, the electrochemical performance of plain and sparked 3D-printed carbon electrodes was evaluated by comparative faradaic EIS measurements in 0.1 M PBS, pH 7, containing 1 + 1 mM potassium hexacyanoferrate (II)/(III). As can be seen from the Bode magnitude plots in Figure 2B, over the high-frequency region (*f* > 10 kHz) where the contribution of any capacitive currents is negligible and the measured impedance corresponds to the total ohmic resistance due to the uncompensated resistance of the electrolyte (*R*_u_), the ohmic resistance of connection cables (*R*_cable_), and the ohmic resistance of the working electrodes,^29^ impedance magnitude decreased as the number of printing layers increased. Considering that, in these measurements, the sum *R*_u_ + *R*_cable_ is constant, impedance values reflect the electric resistance of the electrode. Indeed, impedance values varied following the values of the electrical resistance of the electrodes measured from end to end with a multimeter (Table S2).^24,25^

On the other hand, over the low-frequency range, the sparking process caused a dramatic decrease in the impedance magnitude of the examined electrodes, thus manifesting facile electron transfer at sparked electrodes in comparison with the plain electrodes. Among the examined sparked electrodes, the observed electrocatalytic properties were increased with the increase of the printing layers, in agreement with the CVs in Figure 2A and data in Table S1.

Table S3 compares various reported activation methods in terms of the Δ*E*_p_ values of the activated 3D-printed carbon electrodes, the required time for each method, and whether additional reagents were used for activation. The spark discharge method presented here, in addition to being a reagentless method, exhibits Δ*E*_p_ values among the top-performing methods and is ranked as the fastest among the other methods. These results demonstrate the relevance and the added value of the approach toward the ubiquity of 3D-printed carbon electrodes for electroanalysis.

### Morphological Characterization

**Figure 3:**
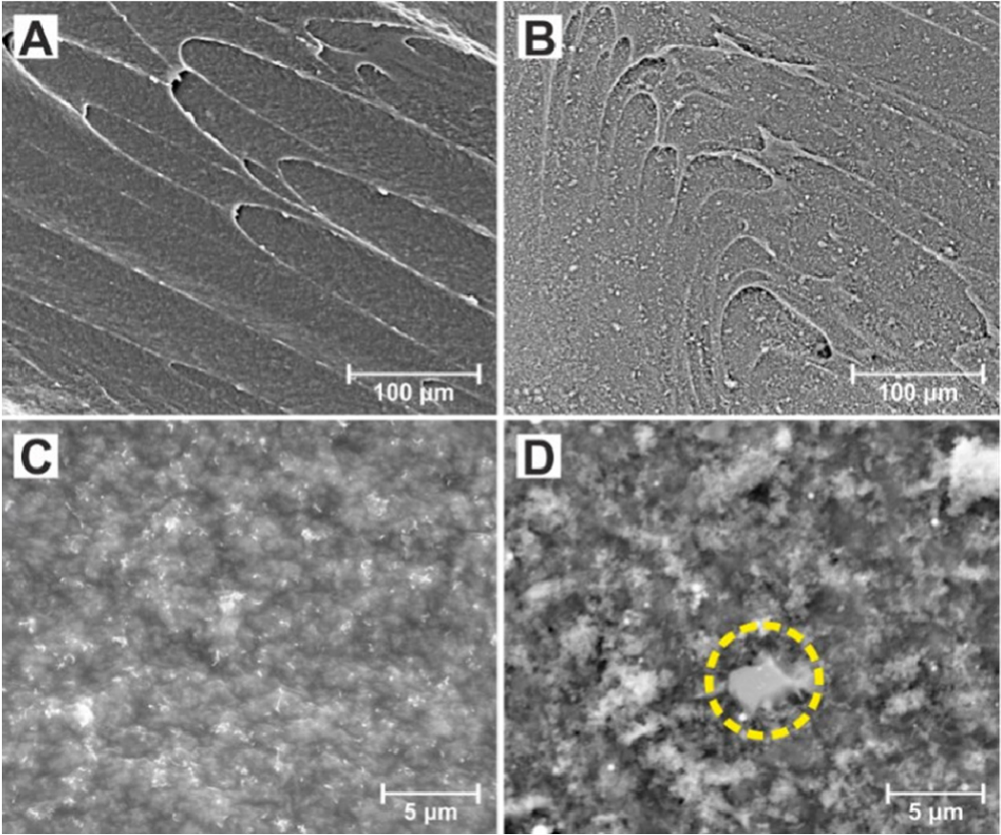
SEM images of (Α,C) plain and (B,D) sparked 3D-printed carbon electrodes at (A,B) lower and (C,D) higher magnification.

Macroscopically, the spark discharge treatment is evident by making the texture of the electrode surface less glossy (data not shown). From the SEM images of plain and sparked 3D-printed carbon electrodes illustrated in Figure 3, it can be inferred that the spark discharge reduced the appearance of molding lines while endowing a grainy texture to the electrode surface (Figure 3A,B). The microscopic inspection of the sparked surfaces at higher magnification (Figure 3D) reveals the existence of sponge-like nanostructures over the entire electrode surface along with the rare appearance of low-dimensional carbon nanostructures (circled area in Figure 3D). For comparison, the morphology of the plain 3D-printed carbon electrode surface at the same magnification is shown in Figure 3C. These morphological and structural alterations at the 3D-printed carbon electrodes’ surface are in accordance with previous studies,^23^ showing that the spark discharge using a graphite pencil electrode tip over a graphite screen-printed electrode results in the formation of such nanodomains.

### ATR-IR and Raman Spectroscopy Characterization

**Figure 4:**
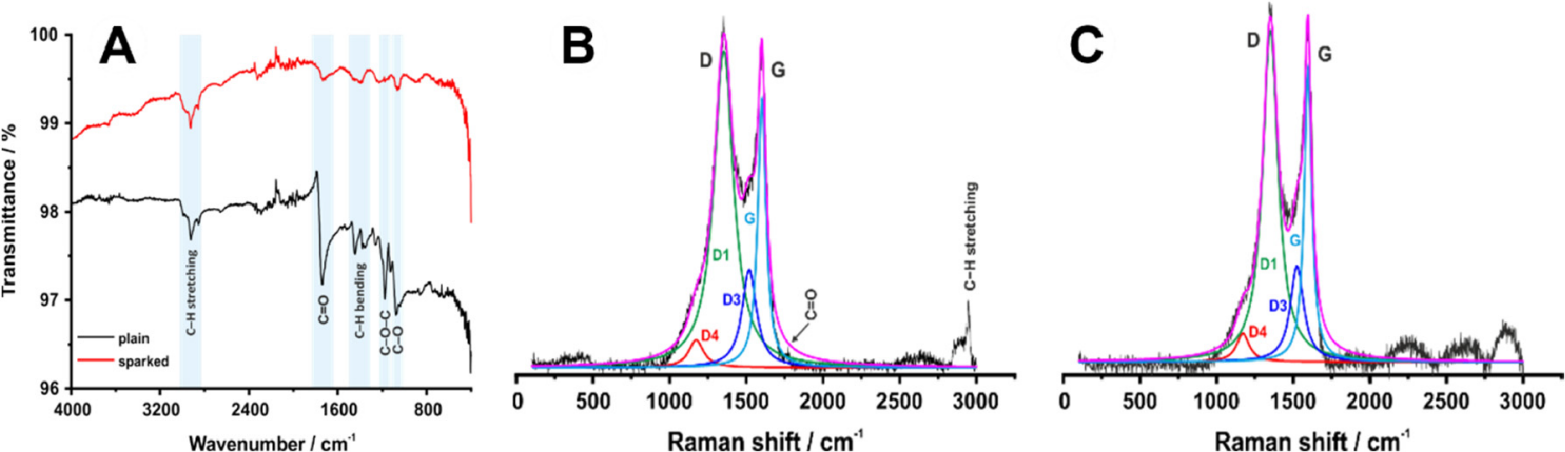
(A) ATR -IR spectra of plain and sparked 3D-printed carbon electrodes. Raman spectra of (B) plain and (C) sparked 3D-printed carbon electrodes.

Figure 4A depicts the ATR-IR spectra of plain and sparked 3D-printed carbon electrodes. The spectrum of a plain 3D-printed carbon electrode displays the characteristic peaks of PLA at 1740 cm^–1^ (C=O), 1175 cm^–1^ (C–O–C), and 1075 cm^–1^ (C–O) related to the structure of the polymeric matrix containing oxygenated functional groups.^18,30−32^ Also, it shows the characteristic bands at 2990 and 2920 cm^–1^, which are assigned to C–H stretching of −CH_3_ asymmetric and −CH_3_ symmetric, respectively, as well as at 1445 and 1370 cm^–1^ that correspondingly are attributed to C–H bending of −CH_3_ asymmetric and −CH_3_ symmetric^18,30−32^ After the treatment by spark discharge, the intensity of all bands decreased significantly, as expected by the removal of PLA from the 3D-printed electrode surface and the exposure of the conductive carbon material.

The Raman spectra of plain (Figure 4B) and sparked (Figure 4C) 3D-printed carbon electrodes are dominated by the D and G bands centered at 1350 and 1595 cm^–1^, respectively. The spectral analysis of these two bands in both cases resulted in a combination of four Lorentzian-shaped bands (G, D1, D3, and D4). Structural changes of plain and sparked 3D-printed carbon electrodes can be studied focusing on two indicators reported in the literature:^23^ (i) *I*_D1_/*I*_G_ ratio, which was employed to demonstrate the existence of defects in the graphitic structure, and (ii) *I*_D3_/*I*_G_ ratio, which expresses the proportion of amorphous carbon in the material.

For the plain 3D-printed carbon electrode, *I*_D1_/*I*_G_ was found to be 1.18, which was slightly decreased to 1.13 for the sparked 3D-printed electrode, showing a slim reduction of defects in the 3D-printed electrodes’ surface after the spark process. The *I*_D3_/*I*_G_ ratio was found to be 0.36 for the plain 3D-printed carbon electrode and 0.31 for the sparked 3D-printed carbon electrode. This implies that the ratio of graphitic domains to amorphous carbon was mildly increased after spark discharge onto the surface of a plain 3D-printed carbon electrode. Moreover, the Raman spectrum of the plain 3D-printed carbon electrode exposes weak bands in the region from 1750 to 1775 cm^–1^, which are attributed to C=O stretch vibrations according to the literature.^33^ More specifically, the band located at 1770 cm^–1^ is characteristic of the amorphous phase of PLA, and it is not observed in the spectrum of the sparked 3D-printed carbon electrode, showing that the PLA has been degraded after the spark discharge. The same conclusion is drawn from the disappearance of the intense band at 2947 cm^–1^ (CH_3_ symmetric stretch) after the spark discharge, which also constitutes a characteristic band for PLA.^33^

### Analytical Performance

3D-printed carbon electrodes activated by spark discharge were also challenged to the simultaneous determination of DP and 5-HT. DP and 5-HT are neurotransmitters that are distributed in the brain and other regions of the neural system. Their simultaneous determination is of great interest due to their coexistence in biological fluids and have implications in neurodegenerative disorders.^34−39^

**Figure 5:**
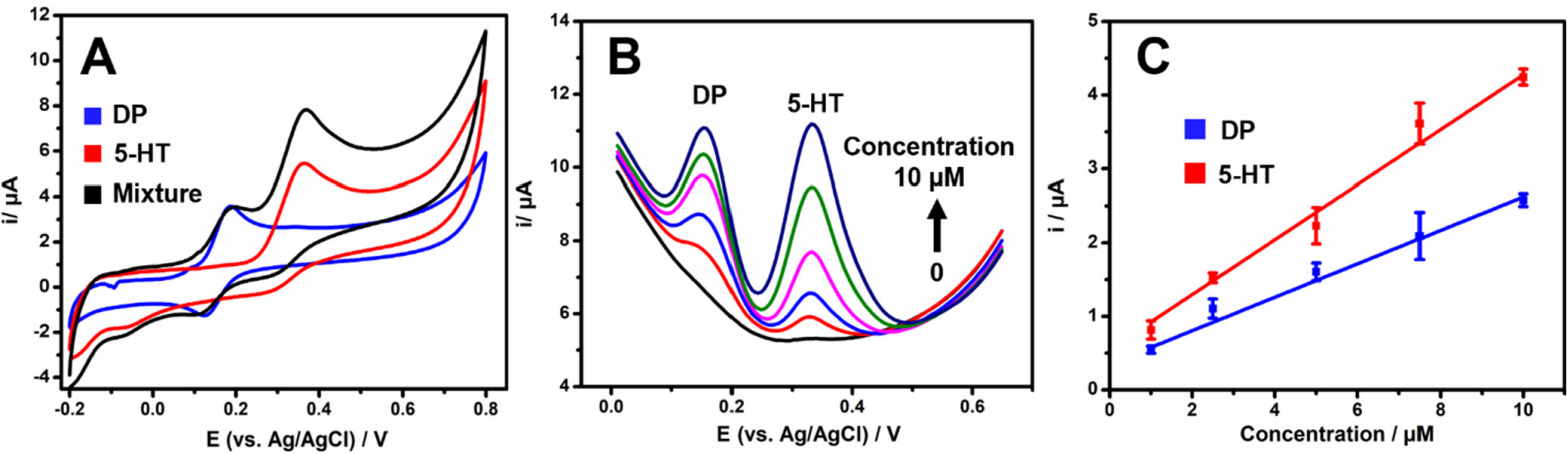
Electrochemical performance of sparked 3D-printed carbon electrodes toward DP and 5-HT. (A) CVs of 100 μM DA (blue), 100 μM 5-HT (red), and an equimolar mixture of DP and 5-HT (black) in 0.1 M PBS, pH 7.0. Scan rate: 0.025 V s^–1^. (B) SW voltammograms on sparked 3D-printed carbon electrodes in 0.1 M PBS, pH 7.0, containing 0, 1, 2.5, 5, 7.5, and 10 μM DP and 5-HT and (C) the respective calibration plots (*n* = 3 devices). Amplitude: 0.05 V, frequency: 5 Hz, and step potential: 0.005 V.

CVs of DP, 5-HT, and an equimolar mixture of them in 0.1 M PBS, pH 7, are shown in Figure 5A. DP exhibited a well-defined oxidation peak at +0.187 V and a reduction peak at +0.127 V. 5-HT displayed an oxidation peak at +0.359 V and a weak reduction peak at +0.288 V. The potential of the oxidation and reduction peaks as well as the peak currents were conserved in the equimolar mixture; hence, the simultaneous determination of both analytes was demonstrated to be possible by employing sparked 10L 3D-printed carbon electrodes.

For the simultaneous DP and 5HT analytical determination, square wave voltammetry was chosen in terms of sensitivity and peak resolution (data not shown). The calibration of each analyte in the presence of the other was studied. First, the calibration features were studied with varying concentrations of both analytes. SW voltammograms of the standard solutions on the sparked 3D-printed electrochemical sensors are shown in Figure 5B. The linear relationship between the peak current from the oxidation peaks of DP and 5-HT and the respective calibration plots over the 1–10 μM concentration range is presented in Figure 5C. The data for DP fitted the equation: *i*_p_(μA) = (0.36 ± 0.06) + (0.23 ± 0.01) [DP] (μM) with a coefficient of determination *R*^2^ = 0.992. In the case of 5-HT, the linear fitting equation was *i*_p_ = (0.59 ± 0.07) + (0.37 ± 0.01) [5-HT] (μM) and a coefficient of determination *R*^2^ = 0.995. The limit of detection (LOD) was determined as 3 *s*/*m* where *s* is the standard deviation of the lowest concentration of the calibration, and *m* is the slope of the straight line. The LODs were 0.6 μM for DP and 0.9 μM for 5-HT.

Next, the calibration features for DP (Figure S1A) and 5-HT (Figure S1B) were repeated but this time fixing the concentration of the other neurotransmitter at 5 μM. In this case, the sensitivity of the method for the DP (in the presence of 5 μM 5-HT) increased in comparison with the individual DP calibration: *i*_p_ (μA) = (0.15 ± 0.05) + (0.36 ± 0.01) [DP] (μM), while for the 5-HT (in the presence of 5 μM DP), the sensitivity remained almost unchanged: *i*_p_ (μA) = (0.88 ± 0.01) + (0.43 ± 0.02) [5-HT] (μM) (Figure S1C). It is important to highlight that the peak current from the electro-oxidation of 5-HT remained constant when the calibration of dopamine was carried out (Figure S1A); however, the peak current for the electrooxidation of dopamine decreased significantly during the 5-HT calibration (Figure S1B).

Then, the intraelectrode and interelectrode repeatability were also studied. Six consecutive SW voltammograms were taken at six different electrodes in the presence of 5 μM DP and 5 μM 5-HT. The results confirmed what was observed in the previous experiments. After the sixth scan, the signal loss for dopamine was (48 ± 6) %, whereas for 5-HT, it was only (10 ± 3) %. This signal loss for DP at carbon electrodes is widely reported and can be minimized by modifying the electrode surface appropriately.^40^ These results agree with the high fouling observed for DP in the presence of 5-HT in calibration studies of the latter. For this reason, intraelectrode repeatability was only studied for 5-HT. The coefficient of variation of the repeatability for six consecutive measurements with the same electrode was found to be 5%. On the other hand, the interelectrode repeatability for 5-HT was found to be 8%, while for DP, it was 7%. In any case, these 3D-printed carbon electrodes are designed to be disposable.

Finally, the 10L electrodes were challenged toward the determination of DP and 5-HT in spiked cell culture medium. Figure S2 displays SW voltammograms for the simultaneous determination of DP and 5-HT in a cell culture, which are summarized in Table S4. Both target analytes were determined with reproducible quantitative recoveries ranging between 95 and 110% (RSDs < 8%) demonstrating the reliability of the spark-activated 3D-printed carbon electrodes for the simultaneous determination of these important neurotransmitters in these media.

On the other hand, the suggested spark discharge activation method does not require preparation of solutions; the electric energy input is negligible and therefore conforms to green analytical chemistry practice.^41^ Various tools were suggested for environmental friendliness evaluation such as GAPI (Green Analytical Procedure Index)^42^ or AES (Analytical Eco Scale).^43^ A recently introduced AGREE score^44^ is accompanied by a free software tool for the greenness evaluation. For the voltammetric determination of neurotransmitters, using the 3D-printed electrochemical cell with a spark-activated electrode surface, the software tool provides the AGREE score of 0.94, i.e., the technique is evaluated as truly green.

## Conclusions

Within the framework of the activation of 3D-printed carbon electrodes as a broad field of current research, herein we have presented a fast and reagentless approach that largely improved the electrocatalytic performance of plain 3D-printed carbon electrodes. First, it was confirmed that, indeed, the electrochemical performance was directly dependent on the thickness of the electrode. Spark discharge treatment improved electron transfer and decreased the total ohmic resistance. From the morphological studies, it was demonstrated that low-dimensional carbon nanodomains were introduced at the electron surface and that the supporting PLA from the filament was degraded after the spark discharge process. The optimized spark-discharge-activated 3D-printed carbon electrodes exhibited good analytical performance and were successfully employed for the simultaneous determination of DP and 5-HT in the cell culture medium. Because of the excellent results obtained as well as the inherent features of the spark discharge reagent-free approach such as direct, fast-speed, and high-frequency discharges; a precisely controlled, ambient-condition automatized process; and a green procedure (0.94 score in GREEnness Metric Approach)^44^ as it involves no solvents or other chemicals, we foresee greater use of this technique to activate filament-based electrodes modifying the electrode surface with different materials. In particular, the full potential of this approach would be attained if the positioning device for the spark-discharge is integrated into the 3D printer and the activation is performed midprint.

## Supporting Information Available

Electrochemical features of spark-activated 3D-printed carbon electrodes extracted from cyclic voltammetry, comparison of electrical resistance between plain and spark-activated 3D-printed carbon electrodes, comparison of the electrochemical parameters among different activation methods for 3D-printed carbon electrodes, dopamine and serotonin individual calibration in a fixed concentration of the other neurotransmitter, and recovery in spiked cell culture (PDF)
