# Supplementary material for: Spark-Discharge-Activated 3D-Printed Electrochemical Sensors
Source: Anal Chem. 2024 Jun 13;96(25):10127–33. doi: 10.1021/acs.analchem.4c01249 (PMC11209655; doi:10.1021/acs.analchem.4c01249)
Supplement: Supplementary file 1 — ac4c01249_si_001.pdf [file ac4c01249_si_001.pdf]

Supporting Information

Spark-Discharge Activated 3D Printed Electrochemical Sensors

Juan F. Hernández-Rodríguez[a,+], Maria G. Trachioti[b,+], Jan Hrbac[c], Daniel Rojas[a,†], Alberto Escarpa*[a,d] and Mamas I. Prodromidis*[b]

[a] *Department of Analytical Chemistry, Physical Chemistry and Chemical Engineering, University of Alcalá, Alcalá de Henares, 28802, Madrid, Spain.*

[b]*Department of Chemistry, University of Ioannina, 45 110 Ioannina, Greece*

[c]*Department of Chemistry, Masaryk University, 625 00 Brno, Czech Republic*

[d] *Chemical Research Institute "Andres M. Del Rio", University of Alcalá, Alcalá de Henares, 28802, Madrid, Spain*

+ These authors contributed equally.

** Corresponding authors*

TABLE OF CONTENT

1. Electrochemical features of spark activated 3D printed carbon electrodes from cyclic voltammetry. 2

2. Comparison of electrical resistance and electrochemical impedance between plain and spark activated 3D-printed carbon electrodes. 3

3. Dopamine and serotonin individual calibration in a fixed concentration of the other neurotransmitter 5

4. Recovery in spiked cell culture medium 6

5. References 7

1. Electrochemical features of spark activated 3D printed carbon electrodes extracted from cyclic voltammetry.

Table S1: Electrochemical results of sparked 3D-printed carbon electrodes made by different number of layers. [a]

Electrode	ΔE_p / V	J / µA cm^{-2}	A / cm^2	k^0 (× 10^{-3}) / cm s^{-1}
1L	0.505 ± 0.046	61.9 ± 4.3	0.075 ± 0.004	0.60 ± 0.02
3L	0.258 ± 0.004	76.8 ± 3.7	0.103 ± 0.006	0.95 ± 0.03
5L	0.220 ± 0.002	81.9 ± 0.6	0.132 ± 0.001	1.05 ± 0.12
8L	0.210 ± 0.003	81.6 ± 3.9	0.150 ± 0.006	1.33 ± 0.01
10L	0.175 ± 0.005	87.1 ± 4.1	0.164 ± 0.006	1.59 ± 0.04

[a] Values correspond to the mean values and the standard deviation for three different electrodes (mean ± SD, n=3). Areas were calculated by using the Randles-Ševčík equation and, the k^o values were calculated by applying the Klingler-Kochi method.[1]

2. Comparison of electrical resistance and electrochemical impedance between plain and spark activated 3D-printed carbon electrodes.

Table S2: Electrical resistance values ($R_{multimeter}$) measured with a multimeter and electrochemical impedance values (Z) at 10 kHz of plain and spark activated 3D-printed carbon electrodes. [a]

Electrode	plain		sparked	
	$R_{multimeter}$ / kOhm	Z / kOhm	$R_{multimeter}$ / kOhm	Z / kOhm
5L	4.8 ± 0.2	4.11 ± 0.04	3.8 ± 0.1	3.59 ± 0.01
8L	3.5 ± 0.2	2.64 ± 0.01	3.0 ± 0.1	2.59 ± 0.01
10L	2.8 ± 0.1	2.05 ± 0.02	2.5 ± 0.1	2.07 ± 0.01

[a]The values correspond to the mean values and the standard deviation for three different electrodes (mean ± SD, n=3).

Table S3. Overview of methods used for the activation of 3D-printed carbon electrodes based on their key electrochemical parameters.

3D-printed electrode	Treatment method	ΔE_p / V [a]	Required time	Reagentless method	Ref.
G-PLA	solvent/electrochemical	0.171	~ 13 min	no	2
G-PLA	electrochemical	0.182	~ 17 min	no	3
CB-PLA	CO_2 laser-ablation/electrochemical	0.450	~ 7 min	no	4
G-PLA	ultrasonication/electrochemical	0.150	~ 1.5 h	no	5
G-PLA	proteinase K-catalyzed digestion	0.180	> 28 h	no	6
CB-PLA	chemical/electrochemical	0.297	~ 7 min	no	7
Gr-GO-PLA	electrochemical	0.370	~ 7 min	no	8
nC-PLA	wet-chemical	0.450	> 24 h	no	9
CNT- CB-PLA	chemical/electrochemical	0.251	> 24 h	no	10
CB-PLA	photochemical/electrochemical	0.182	~ 9 min	no	11
CB-PLA	electrochemical	0.223	~7 min	no	12
CB-PLA	reactive cold oxygen plasma	0.156	2 min	yes	13
CB-PLA	CO_2 laser-scribing	0.130	~ 50 s	yes	14
CB-PLA	He-assisted laser-ablation	0.161[b]	n.m.	yes	15
G-PLA	physical thermal annealing	0.255	> 4 h	yes	16
CB-PLA	electrical (spark) discharge	0.175	~ 30 s	yes	This work

Key: G, graphene; PLA, polylactic acid; CB, carbon black; Gr: graphite; GO, graphene oxide; nC, carbon nanocomposites; CNT, carbon nanotubes. n.m., not mentioned.

[a] Values were calculated using the potassium hexacyanoferrate(II)/(III) redox couple.

[b] Values were calculated using the hexaammineruthenium(III)/(II) trichloride redox couple.

[c] Activation methods were categorized based on whether they are reagentless, with a clear division line separating the two groups

3. Dopamine and serotonin individual calibration in a fixed concentration of the other neurotransmitter

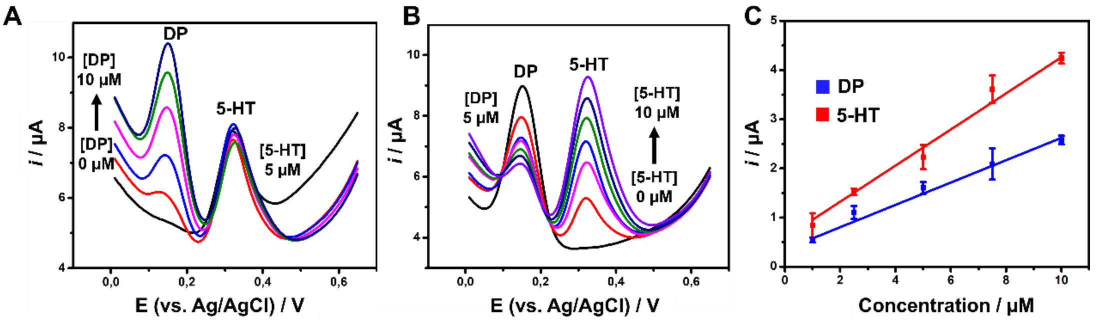

Figure S1. (A) SW voltammograms of DP in the presence of 5 µM of 5-HT, (B) SW voltammograms of 5-HT in the presence of 5 µM of DP, and (C) DP (blue) and 5-HT (red) calibration plots.

4. Recovery in spiked cell culture medium

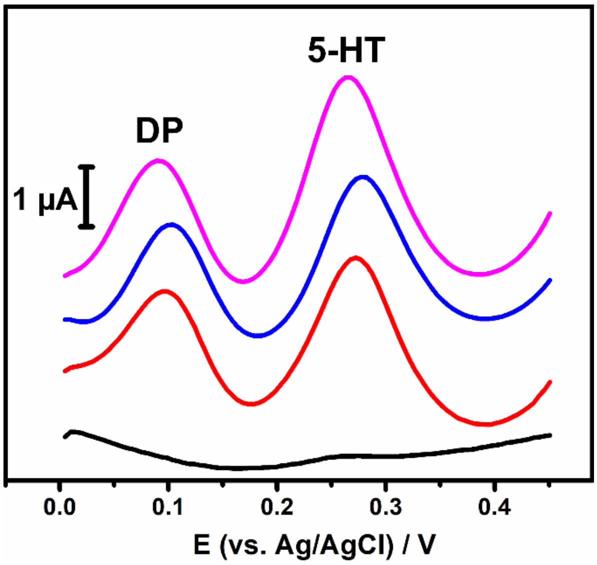

Figure S2. SWV of cell culture media (black) and cell culture media spiked with the following mixtures of DP and 5-HT: 5 μM:5 μM (red), 7.5 μM:5 μM (blue), 7.5 μM:7.5 μM (magenta).

Table S4. DP and 5-HT recovery study in cell culture media (n = 3 3D-printed electrodes).

Amount added (μM)		Amount determined (μM)		Recovery (%)	
DP	5-HT	DP	5-HT	DP	5-HT
2.5	5	2.8 ± 0.4	5.5 ± 0.5	110 ± 10	111 ± 8
5	5	4.9 ± 0.3	5.2 ± 0.2	98 ± 6	106 ± 4
7.5	5	7.3 ± 0.3	5.0 ± 0.3	97 ± 4	100 ± 6
7.5	7.5	7.1 ± 0.2	7.4 ± 0.3	95 ± 3	99 ± 4

5. References

[1] Trachioti, M. G;. Lazanas, A. Ch.; Prodromidis, M. I. *Shedding light on the calculation of electrode electroactive area and heterogeneous electron transfer rate constants at graphite screen-printed electrodes*. Microchim. Acta, 2023, 190 (7), 251.

[2] Browne, M. P.; Novotný, F.; Sofer, Z.; Pumera, M. *3D Printed Graphene Electrodes' Electrochemical Activation.* M. ACS Appl. Mater. Interfaces, 2018, 10 (46), 40294–40301.

[3] dos Santos, P. L.; Katic, V.; Loureiro, H. C.; dos Santos, M. F.; dos Santos, D. P.; Formiga, A. L. B.; Bonacin, J. A. *Enhanced performance of 3D printed graphene electrodes after electrochemical pre-treatment: Role of exposed graphene sheets.* Sens. Actuat. B-Chem, 2019, 281, 837–848.

[4] Veloso, W. B.; Ataide, V. N.; Rocha, D. P.; Nogueira, H. P.; de Siervo, A.; Angnes,L.; Muñoz, R. A. A.; Paixão, T. R. L. C. *3D-printed sensor decorated with nanomaterials by CO2 laser ablation and electrochemical treatment for non-enzymatic tyrosine detection.* Microchim. Acta, 2023, 190 (2), 63.

[5] Grazioli, C.; Svigelj, R.; Dossi, N. *A novel strategy for fabrication, activation and cleaning of fully 3D printed flexible planar electrochemical platforms*. Electroanalysis, 2023, 35 (9), e202300013.

[6] Manzanares-Palenzuela, C. L.; Hermanová, S.; Sofer, Z; Pumera, M. *Proteinase-sculptured 3D-printed graphene/polylactic acid electrodes as potential biosensing platforms: towards enzymatic modeling of 3D-printed structures.* Nanoscale, 2019, 11 (25), 12124–12131.

[7] Rocha, D. P.; Squissato, A. L.; da Silva, S. M.; Richter, E. M.; Munoz, R. A. A. *Improved electrochemical detection of metals in biological samples using 3D-printed electrode: Chemical/electrochemical treatment exposes carbon-black conductive sites.* Electrochim. Acta, 2020, 335, 135688.

[8] J.-H. Shin, K.-D. Seo, H. Park, H. J. Park, and D.-S. Park. *Performance Improvement of Acid Pretreated 3D-printing Composite for the Heavy Metal Ions Analysis*. Electroanalysis, 2021, 33 (7), 1707–1714.

[9] Redondo, E.; Muñoz, J.; Pumera, M. *Green activation using reducing agents of carbon-based 3D printed electrodes: Turning good electrodes to great*. Carbon, 2021, 175, 413–419.

[10] Contreras-Naranjo, J. E.; Perez-Gonzalez, V. H.; Mata-Gómez, M. A.; Aguilar, O. *3D-printed hybrid-carbon-based electrodes for electroanalytical sensing applications*. Electrochem. Commun., 2021, 130, 107098.

[11] de A. Silva-Neto, H.; Santhiago, M.; Duarte, L. C.; Coltro, W. K. T. *Fully 3D printing of carbon black-thermoplastic hybrid materials and fast activation for development of highly stable electrochemical sensors.* Sens. Actuat. B-Chem, 2021, 349, 130721.

[12] Miller, C.; Keattch, O.; Shergill, R. S.; Patel, B. A. *Evaluating diverse electrode surface patterns of 3D printed carbon thermoplastic electrochemical sensors.* Analyst, 2024, 149, 1502-1508.

[13] Pereira, J. F. S.; Rocha, R. G.; Castro, S. V. F.; João, A. F.; Borges, P. H. S.; Rocha, D. P.; de Siervo, A.; Richter, E. M.; Nossol, E.; Gelamo, R. V.; Muñoz, R. A. A. *Reactive oxygen plasma treatment of 3D-printed carbon electrodes towards high-performance electrochemical sensors.* Sens. Actuat. B-Chem., 2021, 347, 130651.

[14] Rocha, D. P.; Ataide, V. N.; de Siervo, A.; Gonçalves, J. M.; Munoz, R. A. A.; Paixão, T. R. L. C.; Agnes, L. *Reagentless and sub-minute laser-scribing treatment to produce enhanced disposable electrochemical sensors via additive manufacture*. Chem. Eng. J., 2021, 425, 130594.

[15] Glowacki, M. J.; Cieslik, M.; Sawczak, M.; Koterwa, A.; Kaczmarzyk, I; Jendrzejewski, R.; Szynkiewicz, L.; Ossowski, T.; Bogdanowicz, R.; Niedzialkowski, P.; Ryl, *Helium-assisted, solvent-free electro-activation of 3D printed conductive carbon-polylactide electrodes by pulsed laser ablation.* J. Appl. Surf. Sci., 2021, 556, 149788.

[16] Novotný, F.; Urbanová, V.; Plutnar, J.; Pumera, M. *Preserving Fine Structure Details and Dramatically Enhancing Electron Transfer Rates in Graphene 3D-Printed Electrodes via Thermal Annealing: Toward Nitroaromatic Explosives Sensing*. ACS Appl. Mater. Interfaces, 2019, 11 (38), 35371–35375.
